# Overcoming the Performance Cliff at High Loadings: Enhanced Mechanical, Dielectric, and Rheological Properties of PBT Composites Reinforced with Flat Glass Fibers

**DOI:** 10.3390/polym18202561

**Published:** 2026-10-17

**Authors:** Zhenhua Fan, Zecong Wei, Qingwen Zeng, Guobing Wei, Weiting Liu, Yong He, Xiaolong Yang, Weidong Xie

**Affiliations:** 1 School of Materials Science and Engineering, Chongqing University, Chongqing 400044, China; 2 Chongqing Polycomp International Co., Ltd., Chongqing 400082, China

**Keywords:** flat glass fiber, PBT, mechanical properties, dielectric properties, rheological properties

## Abstract

High fiber loadings often cause a performance cliff in short-glass-fiber-reinforced thermoplastics because of fiber agglomeration. Herein, polybutylene terephthalate (PBT) composites reinforced with flat glass fibers (FGFR) and conventional circular glass fibers (CGFR) are systematically compared at loadings of 20–50 wt%. The flat fibers exhibit a specific surface area of 0.50 μm^−1^, approximately 1.61 times that of the circular fibers (0.31 μm^−1^). At 50 wt% loading, FGFR achieves a tensile strength of 115.92 ± 1.29 MPa and a notched impact strength of 13.07 ± 2.06 kJ/m^2^, which are 11.4% and 29.7% higher than those of CGFR, respectively (*p* < 0.001 and *p* < 0.05, Welch’s *t*-test). In contrast, CGFR reaches its maximum tensile strength of 107.45 ± 3.84 MPa at 40 wt% and then decreases by 3.2% at 50 wt%; together with a significant quadratic (inverted-U) trend of the CGFR strength–loading curve (*p* < 0.001) and the significant FGFR–CGFR divergence at 50 wt%, this reversal marks the onset of the performance cliff. At 10 GHz and 50 wt% loading, FGFR exhibits a dielectric constant of 4.30 and a loss tangent of 0.014, which are 17.8% and 22.2% lower than those of CGFR, respectively, owing to suppressed interfacial polarization associated with the preferential layered arrangement of the flat fibers. Rheologically, as the fiber content increases from 20 to 50 wt%, the melt flow rate of FGFR decreases by approximately 35.5%, whereas that of CGFR decreases by approximately 60.5%. The modified Halpin–Tsai, Kelly–Tyson, Maxwell–Garnett, and Krieger–Dougherty models, incorporating fiber geometry and a unified agglomeration correction, semi-quantitatively capture the tensile, impact, dielectric, and rheological properties. These results demonstrate that flat-fiber cross-section engineering is an effective strategy for developing high-performance wave-transparent composites under high filler loadings.

## 1. Introduction

The rapid advancement of fifth-generation mobile communications (5G), millimeter-wave radar, and intelligent transportation systems is placing increasing demands on structural materials. These materials must simultaneously exhibit excellent mechanical properties, good processability, and favorable dielectric characteristics-specifically, a low dielectric constant (ε_r_ < 5) and a low dielectric loss tangent (tanδ < 0.02) to minimize electromagnetic energy loss and enable the molding of complex components. The development of high-performance thermoplastic composites has become a key research focus in high-frequency communications and advanced manufacturing [1].

Glass fiber-reinforced thermoplastics are a class of high-performance engineered materials in which the reinforcing phase enhances mechanical strength and impact toughness, while the polymer matrix contributes processability, thermal stability, and dimensional integrity [2,3,4,5,6]. Polybutylene terephthalate (PBT) is widely regarded as one of the ideal materials for electronic packaging and high-frequency communication structural components among many thermoplastic engineering plastics, owing to its low dielectric constant, low dielectric loss, good dimensional stability, and excellent electrical insulation properties [7,8,9]. Owing to the synergistic reinforcement of PBT resin and glass fibers, GF/PBT composites show promise for electronics and electrical engineering, automobile lightweighting, and high-frequency communications [10,11,12,13,14].

Significant progress has been made in the study of glass-fiber-reinforced polybutylene terephthalate (GF/PBT) composites. Hamlaoui et al. [15] systematically examined the role of glass fiber content in determining the mechanical response of PBT composites. Threepopnatkul et al. [16] further demonstrated that both fiber length and content significantly affect flexural properties, albeit with a trade-off in impact resistance. Mörl et al. [17] explored the compressive fracture behavior of glass fiber-reinforced composites and found that microcracking and fiber microbuckling (kink-band formation) serve as energy dissipation mechanisms during fracture propagation. These damage modes, induced by the presence of short fibers in the interlaminar regions, contribute to enhanced energy absorption and improved compressive strength. Fung et al. [18] revealed that injection molding parameters—including injection pressure, melt temperature, and mold temperature—critically influence fiber orientation and distribution uniformity in the part, thereby affecting the effective load-carrying capacity of the fibers and the efficiency of interfacial stress transfer. However, the reinforcement in current industrial GF/PBT composites mostly consists of short-cut glass fibers with a circular cross-section. Circular fibers have a relatively small specific surface area, which limits the interfacial contact area with the matrix. This restricted interface makes the composite particularly susceptible to local stress concentration and interfacial delamination at high fiber loadings, ultimately degrading overall performance and limiting practical applications [19,20,21,22].

Non-circular glass fibers have recently attracted increasing attention as alternative reinforcing materials. Bond et al. [23] reported that triangular glass fibers exhibit a 60% higher single-fiber compressive strength than circular fibers, owing to their larger cross-sectional moment of inertia-demonstrating that fiber geometry directly governs the upper bound of load-bearing capacity. Non-circular fibers are more susceptible to shear failure, allowing their effective length to be controlled during injection molding or extrusion. This leads to increased effective load-bearing efficiency of composites, as demonstrated by Thomason’s theoretical modeling [24] and later confirmed experimentally [21] that the larger specific surface area and interfacial contact area of thin non-circular glass fibers reduce interfacial stress concentration, enabling the material to maintain good structural stability and mechanical consistency at high fiber contents. From a multiphysics perspective, Nakka [25] showed the coupled effect of fiber cross-sectional structure on the thermal, electrical, and magnetic properties of unidirectional composites, indicating that material properties evolve non-monotonically as the shape factor increases, which exemplifies the complexity of geometric control in functional composite design. Thomason et al. [26] provided a systematic analysis of the transition of non-circular glass fibers from laboratory development to industrial applications, highlighting their potential for enhanced dimensional stability, warpage suppression, and interfacial performance. Using non-circular glass fibers in a PBT matrix is a promising route to high-performance wave-transparent composites. Unlike thermoset matrices, thermoplastic PBT composites undergo severe shear heating and fiber attrition during melt processing, and their dielectric and rheological responses at high fiber loadings remain poorly understood for non-circular fiber architectures. Most reported studies on non-circular fibers focus on thermoset matrices; for thermoplastic PBT systems, systematic studies of how fiber cross-section morphology simultaneously governs mechanical, dielectric, and rheological behavior—especially at high fiber contents—remain scarce.

In the present study, flat (non-circular) short glass fibers and conventional circular fibers in a PBT matrix are systematically compared, aiming to establish structure-property relationships across mechanical, dielectric, and rheological domains, particularly at high fiber contents. The key question addressed is how the flat cross-section, through its larger interfacial contact and improved dispersion, overcomes the performance deterioration commonly observed in highly filled circular fiber composites. This work thereby provides both mechanistic understanding and experimental guidance for the design of high-performance wave-transparent thermoplastic composites.

The novelty of this work is threefold. First, to the best of our knowledge, this is the first systematic comparison of flat versus circular short glass fibers in a thermoplastic PBT matrix across the full 20–50 wt% loading range, in which mechanical, dielectric, and rheological responses are resolved simultaneously; previous studies on non-circular fibers have focused mainly on thermoset matrices or on isolated properties. Second, this work demonstrates that fiber cross-section engineering can suppress the agglomeration-induced performance cliff at high loadings: the flat-fiber composite retains monotonic strength growth up to 50 wt% (115.92 MPa), at which the circular-fiber counterpart has already entered the deterioration regime. Third, a shape-dependent modeling framework is established by coupling a unified, geometry-specific fiber-agglomeration correction into the modified Halpin–Tsai/Kelly–Tyson, Maxwell–Garnett, and Krieger–Dougherty models, enabling semi-quantitative prediction of cross-section-shape effects across all three property domains.

## 2. Experimental Section

### 2.1. Raw Materials

Two types of chopped glass fiber/PBT composite pellets were used: flat (non-circular) cross-section pellets (hereafter FGFR; glass fiber grade HL303N-M4) and conventional circular-section pellets (hereafter CGFR; glass fiber grade ECT303K). The flat glass fibers (HL303N-M4) and the conventional circular glass fibers (ECT303K) were both supplied by Chongqing Polycomp International Co., Ltd. (CPIC), Chongqing, China. The flat glass fiber (HL303N-M4) has a rectangular cross-section of approximately 20 μm in width and 5 μm in thickness, corresponding to a cross-sectional aspect ratio of about 4:1. The circular glass fiber (ECT303K) has a nominal diameter of approximately 13 μm. The matrix was a medium-viscosity injection-molding-grade PBT resin (BASF 4300, BASF, Ludwigshafen am Rhein, Germany; equivalent grades from DuPont (Wilmington, DE, USA) and Wanhua Chemical (Yantai, China)were also used in trial batches). The composite pellets were prepared by twin-screw extrusion compounding, and the glass fibers were surface-modified with a silane sizing (coupling agent) to improve the interfacial adhesion with the resin. The sizing of both fiber grades belongs to the supplier’s dedicated sizing platform developed for PBT resin matrices. It is applied as a water-based emulsion (typically 92–97 wt% water) whose organic phase comprises epoxy- and polyurethane-type film formers and an aminosilane coupling system, together with minor auxiliary components (an antistatic agent and a pH regulator); the exact proportions are proprietary to the supplier. Spectroscopic (FTIR, ^1^H NMR) and gel permeation chromatography analysis of the extracted sizing identified the film former as an aromatic polyester of the bisphenol A/1,2-propanediol-adipic acid type, with a number-average molecular weight of approximately 2.0 × 10^4^ (*M_w_*/*M_n_* ≈ 1.6). The coupling system combines a conventional aminosilane (γ-aminopropyltriethoxysilane, KH-550) with a proprietary macromolecular aminosilane, whose longer molecular chains provide a tougher interfacial “bridge” while the conventional silane fills the gaps between them; the amino groups of the silanes can react with the carboxyl end groups of PBT to form covalent bonds across the fiber–matrix interface [27]. Because both fiber grades are sized on the same platform under the same application process, the surface treatment of the two geometries is equivalent by design, ensuring a fair comparison of interfacial adhesion. Since PBT is a thermoplastic resin, no hardener or curing agent is involved during processing; an epoxy-type chain extender was added to reduce the carboxyl end-group content and improve the hydrolysis resistance, together with antioxidants and lubricants, to meet the injection-molding requirements of high-end electrical and electronic components. Experimental formulations are shown in Table 1.

### 2.2. Sample Preparation

**Figure 1:**
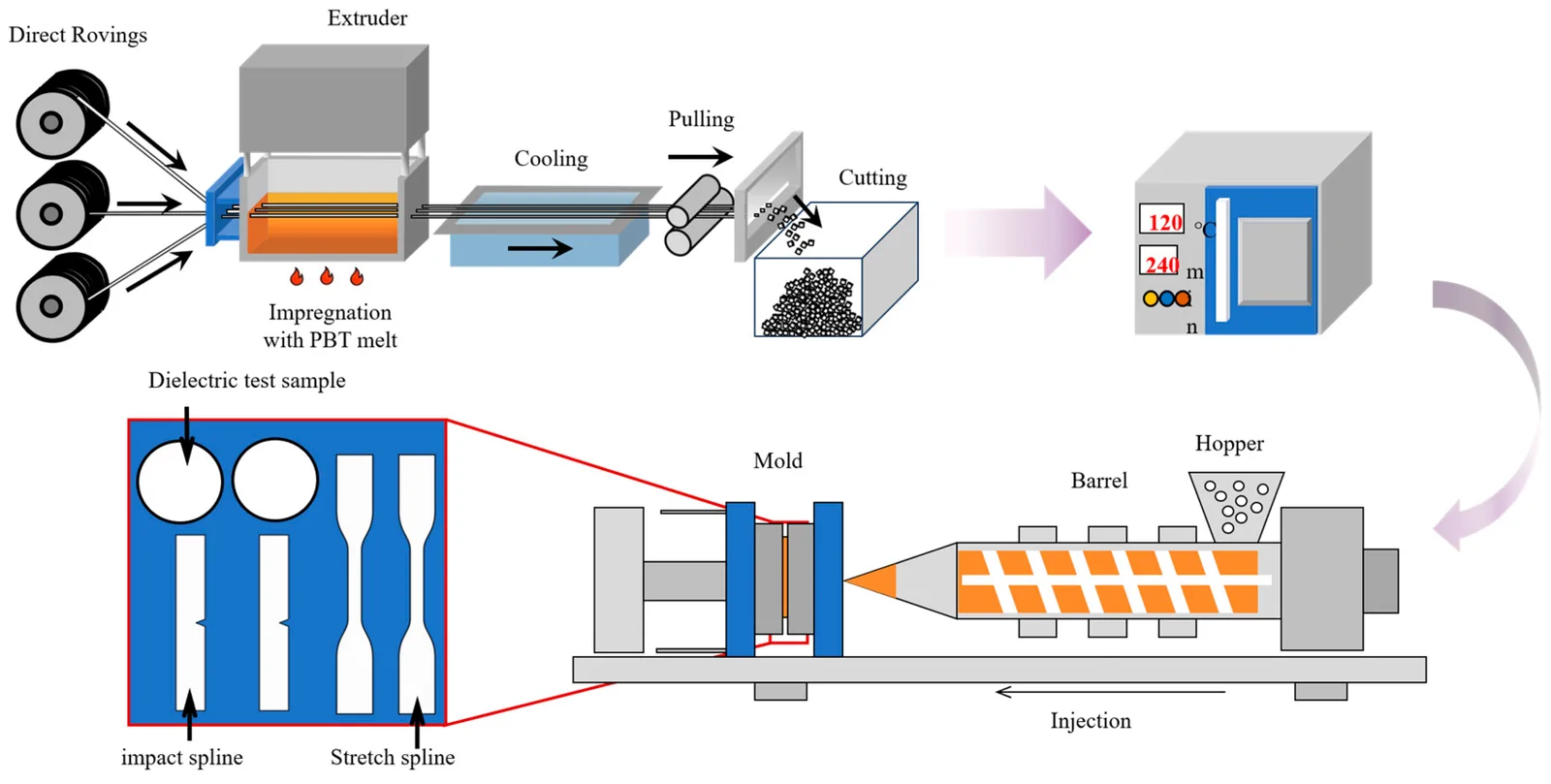
Schematic diagram of the fabrication process for glass-fiber-reinforced PBT composites: glass fiber rovings are impregnated with PBT melt, cooled, pulled, and chopped into composite pellets, which are then dried and injection-molded into test specimens.

Prior to processing, the pellets were dried in a vacuum oven at 120 °C for 4 h. Tensile and impact specimens were subsequently prepared by injection molding. For dielectric testing, the dried pellets were compression-molded using a flat-plate vulcanizer at 260 °C and 10 MPa for 60 min to produce 2 mm-thick sheets. This thermal protocol is identical for both composite systems and remains within the safe processing window of PBT: 260 °C is the upper bound of the recommended melt-processing range of PBT (250–260 °C) [9] and is well below its thermal-decomposition region. Loyer et al. [28] established a time–temperature processing map for PBT, showing that processing-induced degradation proceeds by random chain scission, that oxygen plays a negligible role, and that measurable embrittlement requires markedly harsher conditions (280 °C with a residence time of at least 16 min in an injection-molding barrel), while neither the Young’s modulus nor the yield stress is significantly affected even under the harshest molding conditions. Compression molding is a static, closed-mold process with no shear and minimal oxygen access, and the pellets additionally contain a stabilizing package (antioxidants and an epoxy chain extender that caps carboxyl end groups and rebuilds molecular weight [28]). Consistently, the molded sheets exhibited no discoloration, bubbles, or other signs of degradation, and their dielectric properties fall within the range reported for injection-molded GF/PBT [9]. Because both systems underwent the identical thermal history, any residual thermal effect applies equally to FGFR and CGFR and does not influence the comparative conclusions of this work. The sheets were cut into circular disks (50 mm diameter, 2 mm thickness) using a CNC cutting machine (4060CNC-2.2 kw, Dongguan Jingyan Instrument Technology Co., Ltd., Dongguan, China). The fabrication process of both CGFR and FGFR is shown in Figure 1.

### 2.3. Performance Testing

**Figure 2:**
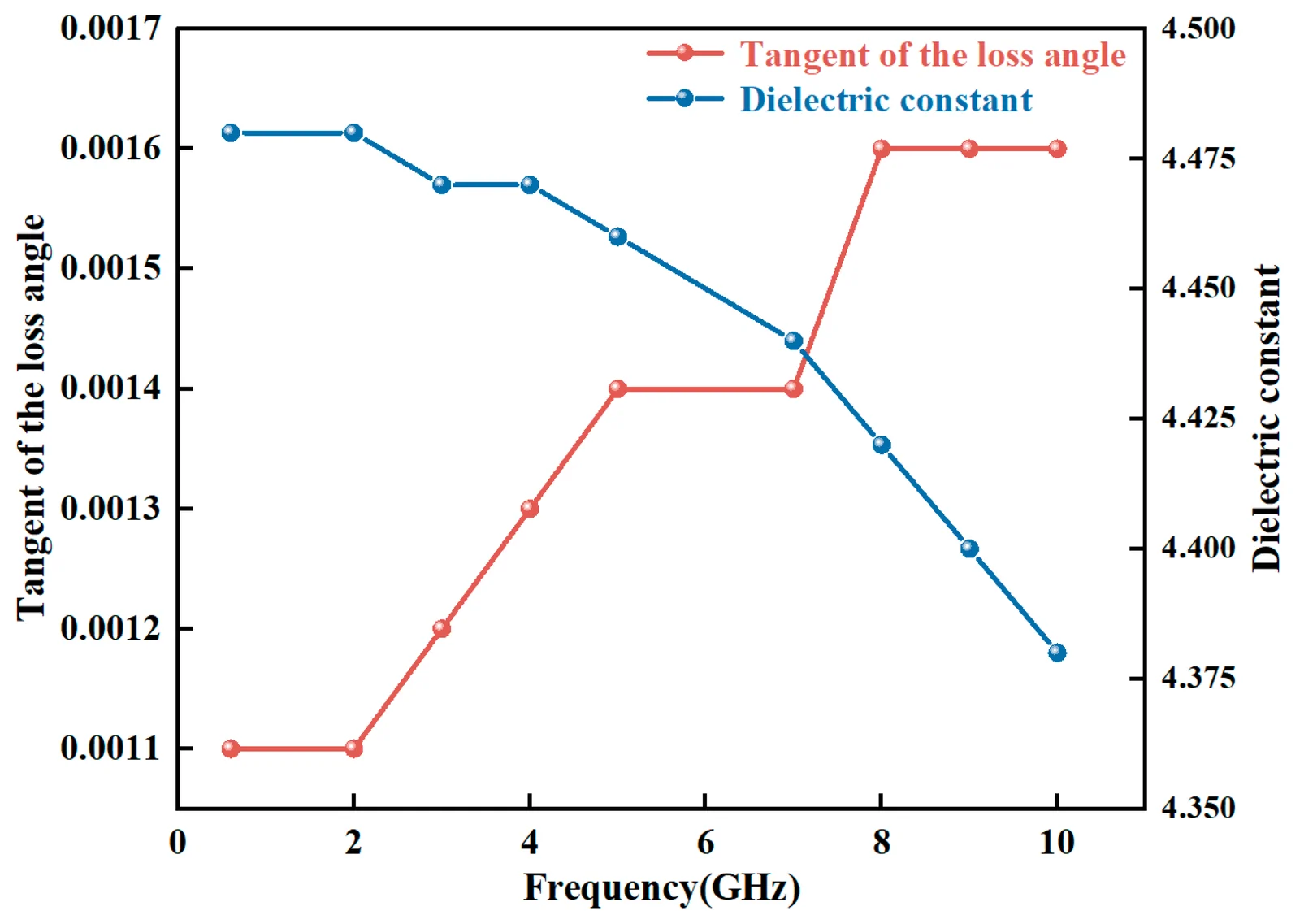
Dielectric constant and dielectric loss tangent for flat glass fibers.

Tensile tests were performed on a universal testing machine (UTM5000, Shenzhen Sansi Zongheng Technology Co., Ltd., Shenzhen, China) at a crosshead speed of 10 mm/min with a grip separation of 115 mm. Five specimens were tested for each formulation. The engineering stress was calculated using the measured cross-section of each specimen, and the strain was derived from the crosshead displacement over the grip separation (apparent strain), with toe compensation applied according to ASTM D638 [29]; the tensile modulus was determined from the initial linear portion (10–50% of the maximum load) of the load–displacement curve. On the toe-compensated curves, this load interval corresponds to apparent strain ranges of 0.12–1.03% (narrowing with increasing fiber content), within which every specimen exhibits excellent linearity (*R*^2^ ≥ 0.990); a chord modulus evaluated between 0.05% and 0.25% apparent strain following the ASTM D638 convention [29] yields the same monotonic trends, with values deviating by less than 15%. Notched impact strength tests were performed using a cantilever beam impact tester (KRT-2022C, Zhejiang Lihong Instrument Co., Ltd., Kunshan, China) in accordance with ASTM D256 [30] (details are listed in Table 2). Injection-molded specimens with dimensions of 84 × 8.3 × 4 mm^3^ (length × width × thickness) were notched on the 84 × 8.3 mm^2^ side face with a notch depth of 2 mm and a notch root radius of 0.25 mm. The specimen was clamped vertically as a cantilever beam, and the pendulum struck the notched face of the specimen. To characterize the residual length and distribution of glass fibers in the composite materials according to GB/T 42923-2023 [31], tensile specimens were placed in crucibles and ashed in a tube furnace (OTF-1200X, Hefei Kejing Materials Technology Co., Ltd., Hefei, China) to remove the PBT matrix, yielding the residual glass fibers. The morphology of the fibers was then observed using a two-photon confocal microscope (Leica TCS SP8 DIVE, Leica Microsystems, Wetzlar, Germany). Statistical analysis of fiber lengths was performed using ImageJ software 1.52t. The dielectric properties were measured using a vector network analyzer (N5227B, Keysight Technologies, Santa Rosa, CA, USA) according to IEC 62562:2010 [32]. For samples tested for dielectric properties, the specimens were first sanded and cleaned with alcohol, then a vector network analyzer was used to measure the dielectric constant and dielectric loss of the high-transparency flat glass fiber-reinforced PBT composite and the control sample at 10 GHz; the dielectric properties of the individual flat glass fibers were also measured over 0.6–10 GHz using the same method; the results are shown in Figure 2. Melt flow index tests were performed on the pellets using a melt flow index tester (Mflow, Zwick Roell, Ulm, Germany) in accordance with GB/T 3682.1-2018 [33], and the mold flow channel length of the melt was measured using an injection molding machine (MA1200III, Haitian Plastic Machinery Group Co., Ltd., Ningbo, China). Finally, the cross-sectional morphology of the tensile and impact test specimens was characterized using a field-emission scanning electron microscope (JSM-7800F, Japan Electron Optics Laboratory Ltd., Tokyo, Japan). Statistical analysis: five specimens were tested for each formulation (*n* = 5) and the data are reported as mean ± SD; pairwise comparisons were performed using Welch’s *t*-test, and loading-dependent trends were assessed by one-way ANOVA followed by Tukey’s HSD post hoc test, with the significance level set at *p* < 0.05; 95% confidence intervals were computed using the *t* distribution (*t*_4_ = 2.776). Analyses were performed with Python 3.11 (SciPy and statsmodels).

## 3. Results and Discussion

### 3.1. Flat Fibers Overcome the Mechanical Performance Cliff at High Loadings

#### 3.1.1. Specific Surface Area and Fiber Length Analysis

For short-fiber glass-reinforced thermoplastic composites, macroscopic mechanical properties are influenced not only by fiber strength, fiber content, and orientation but also by the contact area at the fiber–matrix interface. When the matrix resin type and glass fiber content are held constant, fiber cross-sectional morphology becomes a key factor governing differences in composite mechanical properties [24,34]. Previous studies have shown that glass fibers with flat or non-circular cross-sections possess a higher interfacial area per unit volume, which improves stress transfer behavior between the fibers and the polymer matrix, thereby enhancing the load-bearing capacity and mechanical properties of the composite [24]. Therefore, to quantitatively characterize the geometric differences among glass fibers with different cross-sections, this study introduces specific surface area (SSA) as a structural parameter. For circular-cross-section fibers, the specific surface area per unit length can be expressed as:

(1) $${\mathrm{SSA}}_{\mathrm{circle}}=\frac{\pi d}{\pi d^{2}/4}=\frac{4}{d}$$

**Figure 3:**
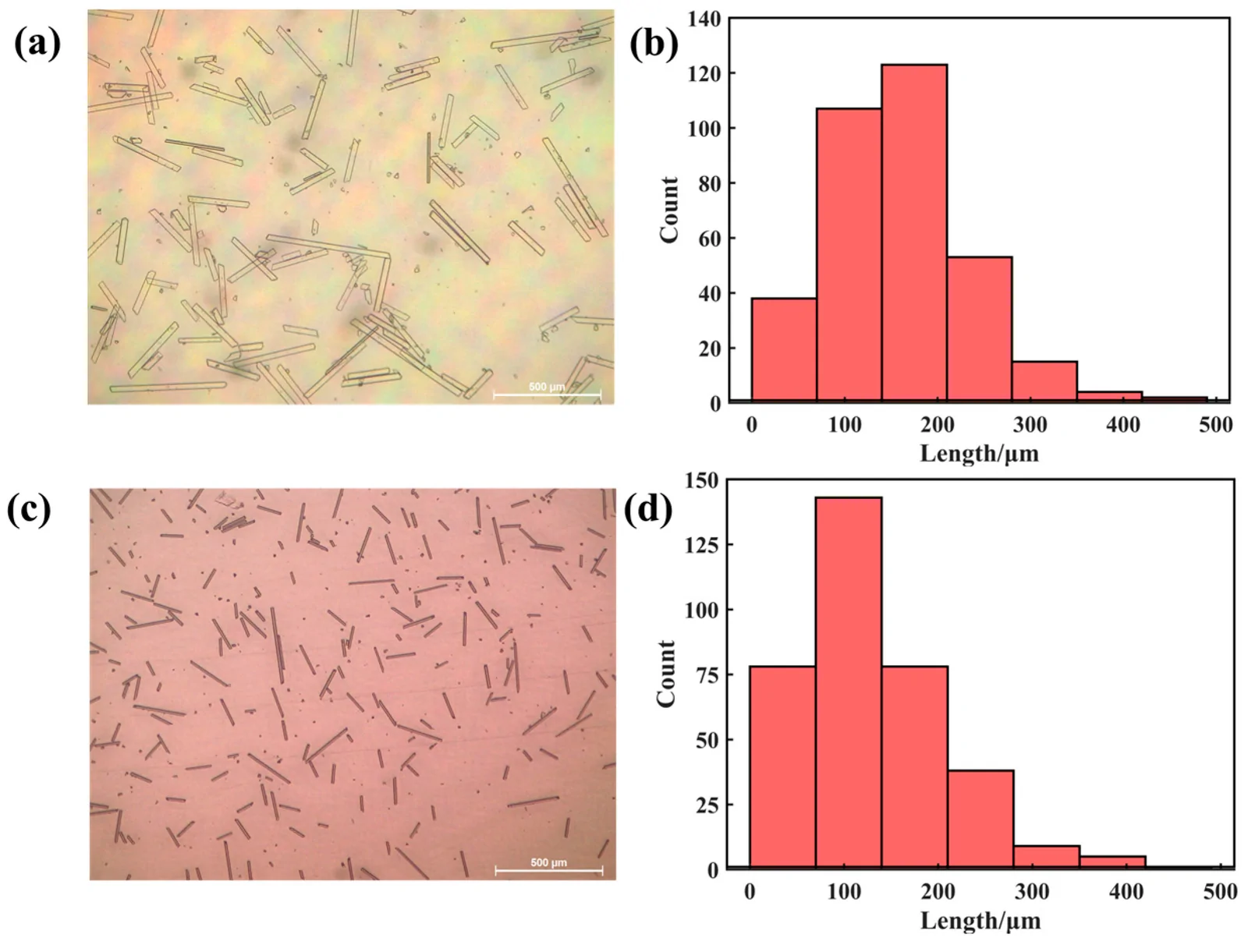
Morphology and length distribution of residual fibers extracted after matrix ashing: (**a**,**c**) optical micrographs of flat and circular fibers, respectively (representative fields used for length measurement); (**b**,**d**) corresponding fiber length distribution histograms.

**Figure 5:**
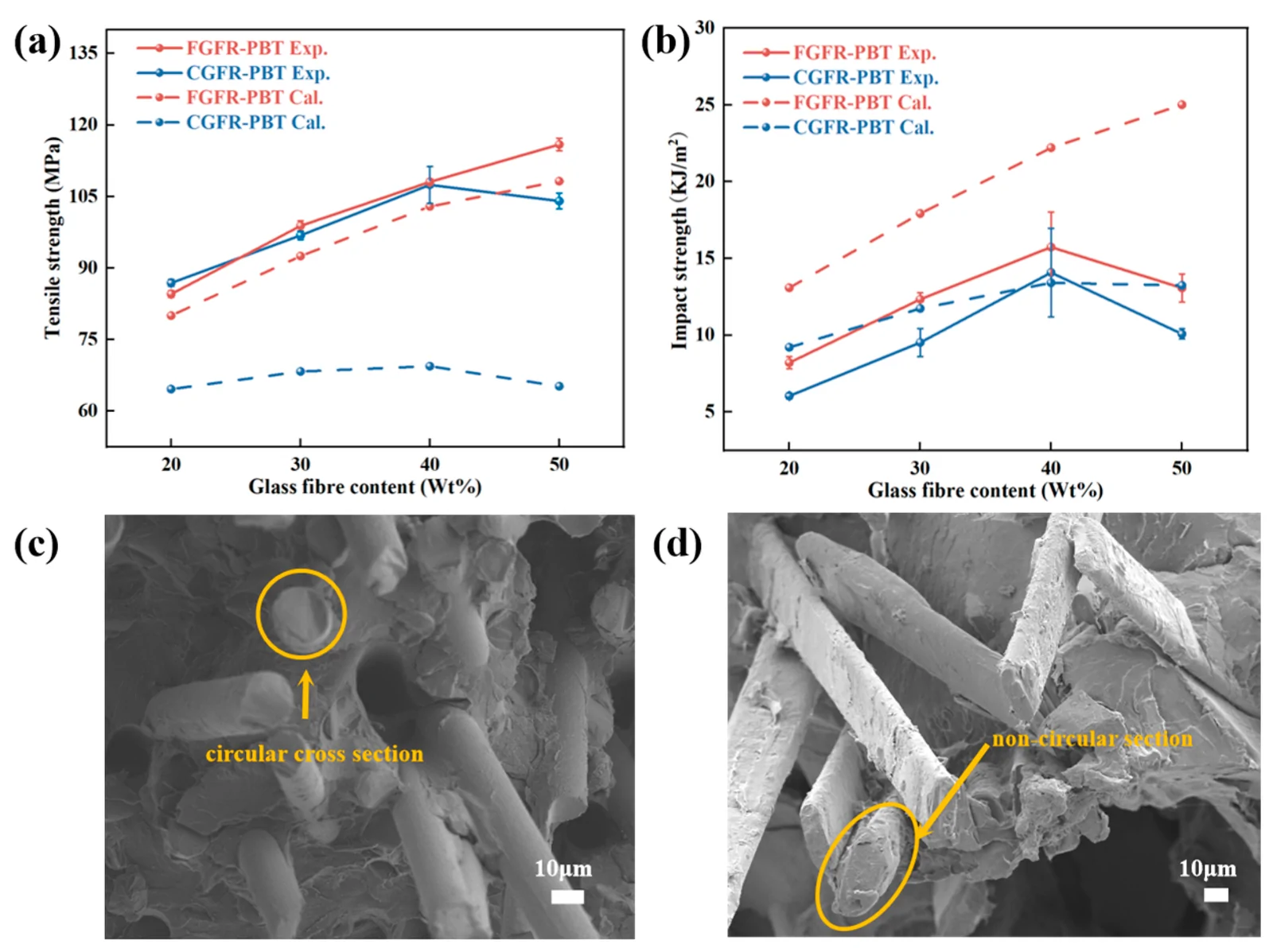
Mechanical properties and fracture morphologies of GF/PBT composites. (**a**) Tensile strength showing the performance cliff of CGFR and its absence in FGFR at 50 wt%; (**b**) Impact strength; (**c**,**d**) SEM images of cross-sections highlighting fiber dispersion differences between circular and flat fibers.

**Figure 6:**
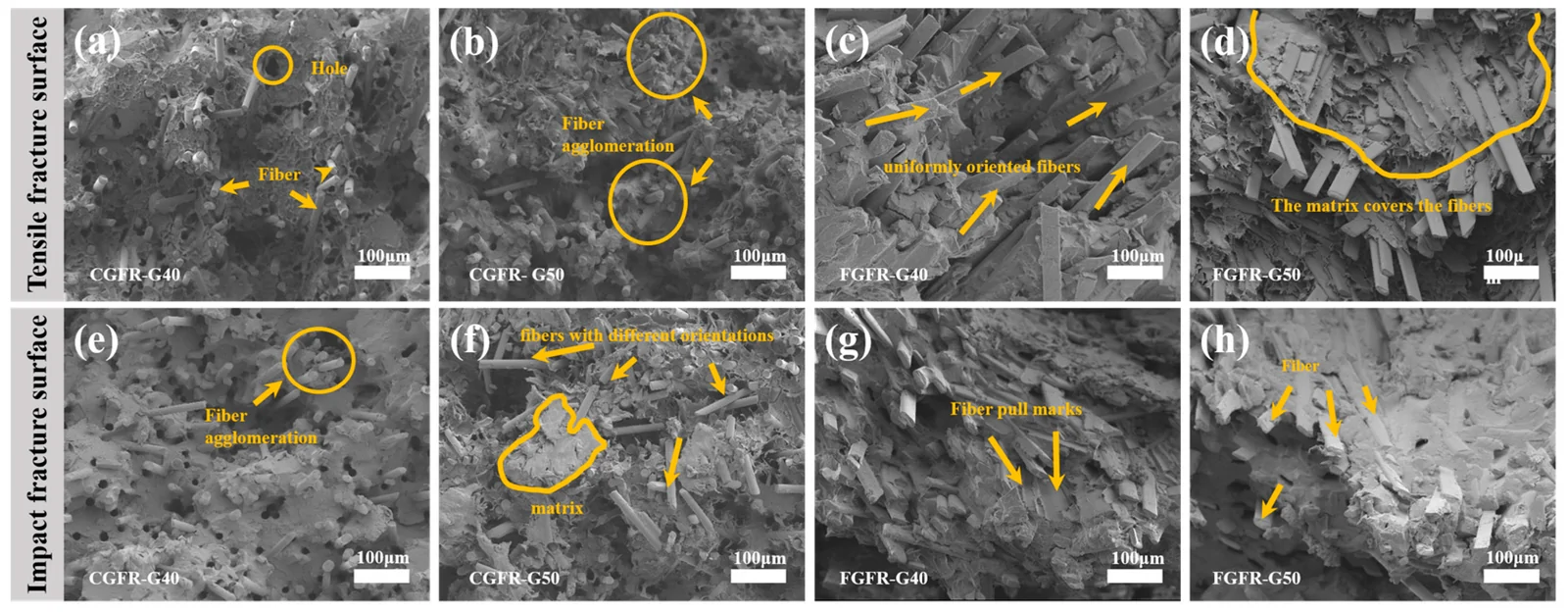
SEM morphology of tensile: (**a**) CGFR-G40; (**b**) CGFR-G50; (**c**) FGFR-G40; (**d**) FGFR-G50 and impact fracture surfaces: (**e**) CGFR-G40; (**f**) CGFR-G50; (**g**) FGFR-G40; (**h**) FGFR-G50.

For flat glass fibers, their specific surface area can be approximated as follows:

(2) $${\mathrm{SSA}}_{\mathrm{non}\text{-}\mathrm{circle}}=\frac{2(w+t)}{\mathrm{wt}}$$

where d represents the diameter of the round glass fiber, while w and t represent the width and thickness of the cross-section of the flat glass fiber, respectively. Specifically, the diameter of the round glass fiber is 13 μm, and the cross-sectional width and thickness of the flat glass fiber are 20 μm and 5 μm, respectively. Calculations show that the specific surface area of the flat glass fiber (0.50 μm^−1^) is 61.3% higher than that of the circular fiber (0.31 μm^−1^), indicating that the flat glass fibers can form a larger interfacial contact area with the PBT matrix, thereby enhancing the efficiency of interfacial load transfer under the same volume fraction conditions. Therefore, theoretically, the mechanical properties of PBT composites reinforced with flat glass fibers should be superior to those of systems reinforced with circular glass fibers. Fiber length tests, as shown in Figure 3a–d, indicate that the average length of flat glass fibers is 156 μm, while that of circular glass fibers is 131 μm. Note that Figure 3 presents residual fibers collected after ashing for length statistics, rather than the in situ dispersion state within the matrix. Because all eight formulations were compounded with the same twin-screw configuration and molded under the identical processing window, the residual fiber length is governed primarily by the fiber cross-section and the initial cut length and varies only weakly with the fiber loading in this range [35,36]; the measured distributions (Figure 3b,d) are therefore used as representative inputs for both systems, and loading-resolved fiber-length mapping is identified as future work. The dispersion uniformity within the composites was verified by SEM observations of multiple cross-sectional regions (Figure 5c,d and Figure 6), which show that the flat fibers remain uniformly dispersed in the PBT matrix even at 50 wt%, whereas the circular fibers exhibit visible entanglement and agglomeration (Figure 5b).

#### 3.1.2. Tensile Strength: Linear Increase Without Degradation

**Figure 4:**
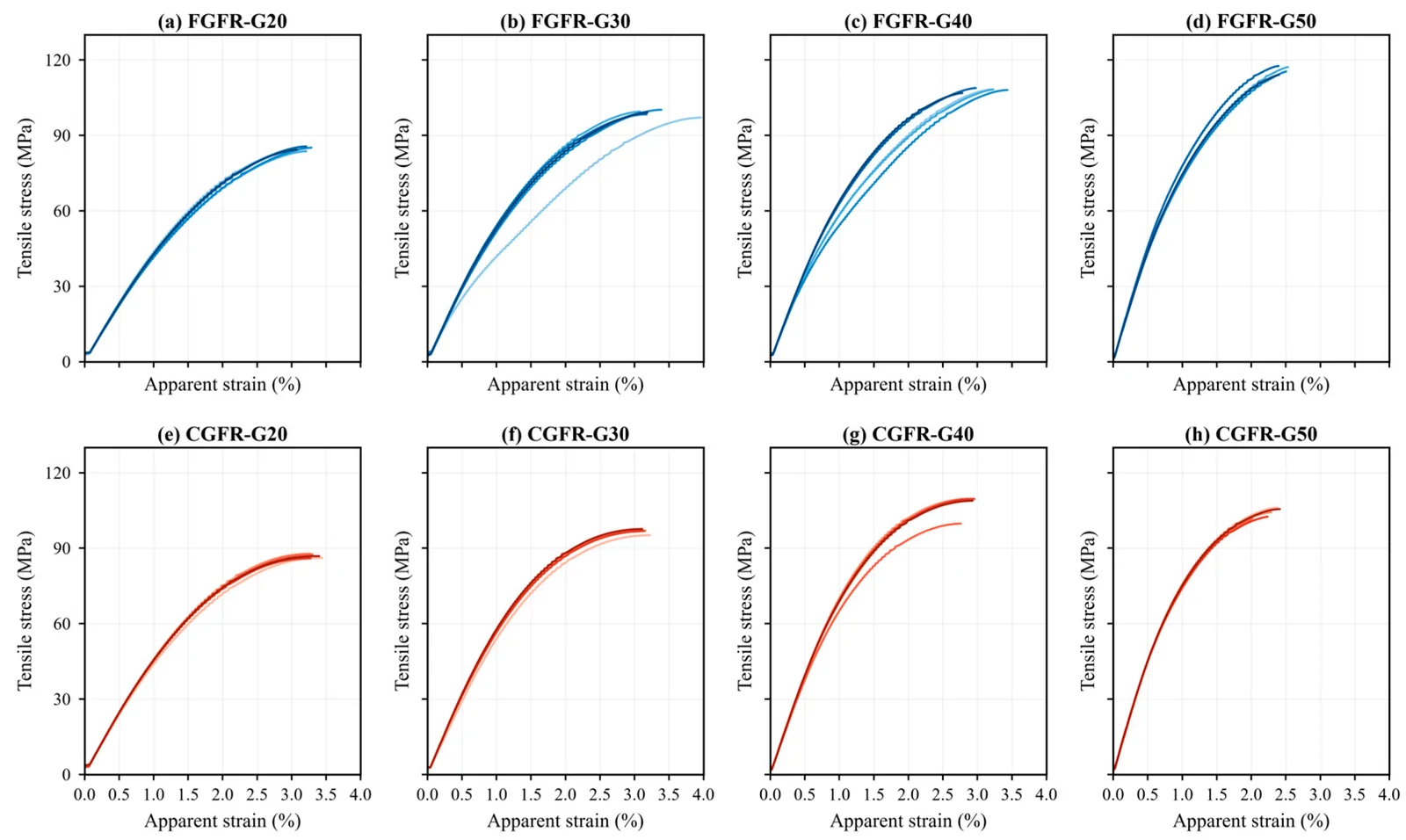
Tensile stress–apparent strain curves of the FGFR and CGFR composites: (**a**) FGFR-G20; (**b**) FGFR-G30; (**c**) FGFR-G40; (**d**) FGFR-G50; (**e**) CGFR-G20; (**f**) CGFR-G30; (**g**) CGFR-G40; (**h**) CGFR-G50. Five parallel specimens are shown for each formulation.

The tensile stress–apparent strain curves of all specimen sets are presented in Figure 4. After toe compensation, all curves are nearly linear up to final fracture, confirming the brittle failure behavior of both composite systems, and the close overlap of the five parallel specimens for each formulation demonstrates satisfactory test repeatability. The apparent tensile moduli of all specimen sets, determined from the initial linear portion (10–50% of the maximum load, corresponding to apparent strains of 0.12–1.03% with *R*^2^ ≥ 0.990 for every specimen; see Section 2.3) of the load–displacement curves, are summarized in Table 4. The modulus of both systems increases monotonically with fiber content, rising from 4.17 to 8.06 GPa for the FGFR composites and from 4.46 to 8.55 GPa for the CGFR composites as the fiber content increases from 20 to 50 wt%. Interestingly, the CGFR composites exhibit a slightly higher modulus than the FGFR composites at every fiber loading (by approximately 6–22%), in contrast to the tensile-strength trend at high loadings. This difference reflects the distinct physical origins of the two properties: the modulus is a small-strain property governed primarily by the fiber volume fraction and orientation, whereas the strength is a failure property governed by interfacial load transfer, where the larger specific surface area of the flat fibers provides the decisive advantage. As all specimens were tested under identical conditions, the comparative trends are unaffected by machine compliance, which shifts the absolute values of all groups equally.

Figure 5a depicts the variation in tensile strength of GF/PBT composites with glass fiber content; the corresponding mean values and standard deviations (*n* = 5 for each formulation) are summarized in Table 3. For FGFR, the tensile strength increases monotonically from 84.54 ± 0.71 MPa at 20 wt% to 98.86 ± 1.06 MPa at 30 wt%, 108.05 ± 0.66 MPa at 40 wt%, and 115.92 ± 1.29 MPa at 50 wt%, corresponding to stepwise increments of 16.9%, 9.3%, and 7.3%, respectively, and the final 40→50 wt% increment remains statistically significant (*p* < 0.001, Welch’s *t*-test), confirming that the FGFR strength has not saturated within the investigated range. Pairwise Welch’s *t*-tests between the two systems at each loading show that CGFR is slightly but significantly stronger than FGFR at 20 wt% (*p* = 0.002), that FGFR is marginally higher at 30 wt% (*p* = 0.02), and that the two systems are statistically indistinguishable at 40 wt% (*p* = 0.77). Beyond 40 wt%, the two curves diverge markedly: FGFR retains a quasi-linear increase and reaches its maximum at 50 wt%, whereas CGFR reaches its strength peak of 107.45 ± 3.84 MPa at 40 wt% and then declines by 3.2% to 104.06 ± 1.68 MPa at 50 wt%, so that FGFR exceeds CGFR by 11.4% at 50 wt% (*p* < 0.001). It should be noted that the 3.2% decrease in CGFR between 40 and 50 wt% is not, by itself, statistically significant (*p* = 0.16); the onset of the performance cliff is therefore identified from converging evidence rather than from this single pairwise comparison: (i) the significant divergence between the two systems at 50 wt% (11.4%, *p* < 0.001); (ii) the continued significant increase in FGFR from 40 to 50 wt% (+7.3%, *p* < 0.001); (iii) a significant quadratic (inverted-U) contrast of the CGFR strength–loading curve (*p* < 0.001), which changes the sign of the strength increment, whereas the FGFR increments remain significantly positive throughout; and (iv) a significant increase in data scatter at CGFR-G40 relative to the other CGFR groups (variance-ratio test, *p* < 0.05), which signals the onset of agglomeration-induced instability.

**Table 3:** Tensile strength and notched Izod impact strength of GF/PBT composites (mean ± SD, *n* = 5).

Glass Fiber Content	FGFR Tensile Strength (MPa)	CGFR Tensile Strength (MPa)	FGFR Impact Strength (kJ/m^2^)	CGFR Impact Strength (kJ/m^2^)
20 wt%	84.54 ± 0.71	86.86 ± 0.74	8.20 ± 0.88	6.04 ± 0.43
30 wt%	98.86 ± 1.06	96.84 ± 0.88	12.33 ± 0.96	9.52 ± 2.04
40 wt%	108.05 ± 0.66	107.45 ± 3.84	15.74 ± 5.10	14.08 ± 6.44
50 wt%	115.92 ± 1.29	104.06 ± 1.68	13.07 ± 2.06	10.08 ± 0.75

Note: 95% confidence intervals (*t*_4_ = 2.776). Tensile strength at 20/30/40/50 wt%: FGFR [83.56, 85.52], [97.38, 100.34], [107.14, 108.96], [114.13, 117.71] MPa; CGFR [85.83, 87.89], [95.62, 98.05], [102.13, 112.77], [101.72, 106.40] MPa. Impact strength at 20/30/40/50 wt%: FGFR [7.11, 9.30], [11.14, 13.53], [9.41, 22.07], [10.52, 15.63] kJ/m^2^; CGFR [5.51, 6.57], [6.99, 12.05], [6.08, 22.08], [9.15, 11.00] kJ/m^2^.

**Table 4:** Apparent tensile modulus of the FGFR and CGFR composites (mean ± standard deviation, *n* = 5).

Formulation	Apparent Tensile Modulus (GPa)
FGFR-G20	4.17 ± 0.11
FGFR-G30	4.95 ± 0.81
FGFR-G40	5.93 ± 0.62
FGFR-G50	8.06 ± 0.27
CGFR-G20	4.46 ± 0.08
CGFR-G30	5.76 ± 0.16
CGFR-G40	7.25 ± 0.15
CGFR-G50	8.55 ± 0.08

Note: The apparent strain intervals corresponding to the 10–50% maximum-load fitting window (after toe compensation) are 0.20–1.02%, 0.21–1.03%, 0.18–0.92%, and 0.14–0.72% for FGFR-G20 to FGFR-G50, and 0.19–0.97%, 0.17–0.84%, 0.15–0.74%, and 0.12–0.61% for CGFR-G20 to CGFR-G50, respectively, with *R*^2^ ≥ 0.990 for every specimen.

To further investigate the underlying mechanisms behind the differences in the tensile properties of glass fiber-reinforced PBT composites, this study employed the modified Halpin–Tsai and Kelly–Tyson coupled models to predict the tensile strength of the composites. Since glass fibers are inevitably subjected to shear forces and fracture during the injection molding process, the actual fiber length is typically significantly shorter than the initial cut length [35]; therefore, classical continuous fiber reinforcement theory is no longer sufficient to accurately describe the mechanical behavior of short-glass-fiber-reinforced thermoplastic composites. Accordingly, this study combines the Kelly–Tyson critical fiber length theory with the Halpin–Tsai short-fiber reinforcement theory [34,37] to establish a tensile strength prediction model applicable to short-glass-fiber-reinforced PBT composites, expressed as follows:

(3) $$\sigma_{c}=\chi_{1}\chi_{2}\sigma_{f}V_{f}+\sigma_{m}(1-V_{f})$$

where *σ_c_* represents the tensile strength of the composite material in MPa; *σ_f_* and *σ_m_* represent the tensile strengths of the glass fibers and the PBT matrix, respectively; *V_f_* represents the fiber volume fraction; *X*_1_ is the fiber length efficiency factor, used to characterize the load-transfer efficiency of finite-length fibers; and *X*_2_ is the fiber orientation efficiency factor, used to correct for the reinforcement loss resulting from the random orientation of fibers during the injection molding process. Since the experimental formulations are given in mass fraction, the mass fraction must first be converted to volume fraction as follows:

(4) $$V_{f}=\frac{\rho_{m}W_{f}}{\rho_{m}W_{f}+\rho_{f}(1-W_{f})}$$

where *W_f_* represents the mass fraction of glass fibers; *ρ_m_* and *ρ_f_* represent the densities of the PBT matrix and the glass fibers, respectively. The densities used were 1.31 g/cm^3^ for PBT and 2.54 g/cm^3^ for glass fiber [15]. Based on the above formula, the volume fractions corresponding to fiber contents of 20 wt%, 30 wt%, 40 wt%, and 50 wt% were calculated to be 0.114, 0.181, 0.256, and 0.340, respectively. According to the Kelly–Tyson theory [38], the critical length of a circular fiber can be expressed as:

(5) $$L_{c}=\frac{\sigma_{f}d}{2\tau_{i}}$$

For flat fibers, the critical length follows from the same axial force balance, without invoking any equal-cross-sectional-area assumption: equating the fiber fracture load, *σ_f_*·*w*·*t* (where *w* = 20 μm and *t* = 5 μm are the width and thickness of the flat cross-section [21]), to the interfacial shear force acting over the perimeter of the critically embedded fragment, *τ_i_*·2(*w* + *t*)·(*L_cf_*/2), directly yields [24,39]:

(6) $$L_{cf}=\frac{\sigma_{f}wt}{\tau_{i}(w+t)}$$

Here, *d* is the diameter of the circular fiber, *τ_i_* is the interfacial shear strength, *L_c_* and *L_cf_* are the critical lengths of the circular and flat fibers, and *w* and *t* are the width (20 μm) and thickness (5 μm) of the flat cross-section [21]. For the circular glass fibers, the diameter is 13 μm [21], the interfacial shear strength between the glass fibers and the PBT matrix is 19.3 MPa [21], and the tensile strength of the fibers (*σ_f_*) is 1500 MPa. Substituting these parameters into Equations (5) and (6) yields critical lengths of 505.18 μm for the circular fibers and 310.88 μm for the flat fibers. The corresponding cross-sectional shape factor, now defined by the critical-length ratio *C_f_* = *L_cf_*/*L_c_* = 2*wt*/[*d*(*w* + *t*)], is 0.615, which replaces the previously assumed empirical value of 0.75 [24] and is used consistently throughout this work.

Based on the relationship between the average fiber length $\bar{L}$ and the critical length *L_c_*, the fiber length efficiency factor *X*_1_ can be expressed as:

(7) $$\chi_{1}=\left\{\begin{array}{cc} \frac{\bar{L}}{L_{c}} & \bar{L}<L_{c}\\ 1-\frac{L_{c}}{2\bar{L}} & \bar{L}\geq L_{c} \end{array}\right.$$

Since the average lengths of both circular and flat glass fibers are less than their respective critical fiber lengths, calculations based on the fiber length efficiency factor formula yield fiber length efficiency factors of 0.259 and 0.502 for the circular and flat glass fiber systems, respectively. For short-fiber composites produced by injection molding, the fibers typically exhibit a distinct two-dimensional in-plane random orientation. Under two-dimensional random orientation conditions, the theoretical orientation efficiency factor (*X*_2_) is 0.375 [40,41]. It is emphasized that *X*_2_ = 0.375 refers specifically to the in-plane orientation distribution. The SEM observations (Section 3.1) show that the flat fibers lie with their wide faces preferentially parallel to the specimen surface, i.e., an out-of-plane layered arrangement, while their in-plane orientation remains essentially random, so that the 2D-random value *X*_2_ = 0.375 applies exactly to the FGFR system. The circular fibers, in contrast, are partially aligned along the flow direction by the converging flow at the mold gate (skin–core structure), so their true in-plane orientation factor slightly exceeds 0.375; adopting 0.375 for the CGFR system is therefore a conservative choice, whose consequences are discussed together with the model predictions below. Substituting the above parameters into Equation (3) yields the baseline tensile-strength predictions. To account for the progressive fiber–fiber interactions that develop with increasing loading in both systems, an agglomeration factor (*η_a_*), combining the maximum fiber volume fraction (*V_fmax_*) with an empirical exponent (*m*), was introduced for both systems and at all loadings [42,43]:

(8) $$\eta_{a}=1-{\left(\frac{1}{V_{\mathrm{fmax}}}\cdot V_{f}\right)}^{m}$$

In particular, the value of *V_fmax_* was calculated using the model proposed by Evans and Gibson [44] for randomly oriented short-fiber composites, which gives:

(9) $$V_{f,\max}=k\frac{d}{L}$$

where *V_fmax_* is the maximum fiber volume fraction, *k* is an empirical constant, *d* is the characteristic lateral dimension of the fiber cross-section, and *L* is the average fiber length. Given that *k* = 5.3 [44], substituting the diameter (13 μm) and the average fiber length (131 μm) into Equation (9) yields *V_fmax_* = 0.526 for the circular fibers; for the flat fibers, face-to-face stacking is governed by the cross-section width, and substituting the width (20 μm) and the average fiber length (156 μm) yields *V_fmax_* = 0.68. With an empirical exponent of *m* = 2 [45], the agglomeration factor *η_a_* = 1 − (*V_f_*/*V_fmax_*)^2^ evaluates to 0.953, 0.882, 0.763, and 0.582 for the CGFR system and 0.972, 0.929, 0.858, and 0.750 for the FGFR system at 20, 30, 40, and 50 wt%, respectively; the value for the CGFR system at 50 wt% (0.582) is identical to that used in our previous analysis. After taking the fiber agglomeration effect into account, the corrected model for the tensile strength of composite materials can be expressed as:

(10) $$\sigma_{c}=\eta_{a}x_{1}x_{2}\sigma_{f}V_{f}+\sigma_{m}\left(1-V_{f}\right)$$

The agglomeration correction is applied uniformly to both systems at all loadings, with the geometry-specific *V_fmax_* values given above. The predicted tensile strengths are 64.6, 68.3, 69.4, and 65.2 MPa for the CGFR composites and 80.0, 92.5, 102.9, and 108.2 MPa for the FGFR composites at 20, 30, 40, and 50 wt%, respectively; they are plotted as dashed lines in Figure 5a. For the FGFR system, the predictions deviate from the experimental values by only 4.8–6.7% across the whole loading range; i.e., the model is nearly quantitative for the flat-fiber composites, for which the 2D-random orientation factor applies exactly. The CGFR predictions are lower than the measured strengths (e.g., 65.2 versus 104.1 MPa at 50 wt%), which is attributed primarily to the conservative orientation factor: as discussed above, partial flow alignment of the circular fibers raises the true *X*_2_ above the 2D-random value of 0.375, and the consistently higher experimental modulus of the CGFR composites at every loading (Table 4) independently supports a higher effective orientation efficiency of the circular fibers. Additional deviations arise from fiber breakage, residual agglomeration not captured by the mean-field correction, interfacial defects, and the use of number-average fiber lengths. The model should therefore be regarded as a semi-quantitative tool: it reproduces the experimentally observed trends—monotonic strength growth for FGFR versus a strength maximum followed by a decline for CGFR—and correctly ranks the two geometries, confirming that flat glass fibers enhance composite strength through their larger interfacial contact area, more efficient load transfer, and higher packing limit.

The tensile strength curves and SEM morphology (Figure 5) indicate that the incorporation of flat glass fibers significantly improves the tensile strength of PBT composites, with the reinforcing effect being more pronounced at 40 wt% fiber content. Comparison between Figure 5a–d indicates that the failure mechanism within the composite changes dramatically with the increase in the glass fiber content. In the CGFR composite, high fiber loading readily induces a “bridging” effect between fibers, leading to agglomeration and localized stress concentration. This uneven distribution not only reduces stress transfer efficiency but also promotes rapid and continuous crack propagation along the weak fiber–matrix interface. For FGFR, the reinforcing effect of flat glass fibers is superior to that of circular glass fibers at high loadings, owing to the advantages of their distinctive cross-sectional shape. The flat structure increases the effective contact area with the PBT matrix and enhances interfacial bonding through geometric mechanical interlocking. Consequently, even at high loadings, the flat fibers maintain good orientation and dispersion within the matrix. Under loading, the closely bound flat glass fibers effectively induce fracture deflection, pinning, and crack arrest, rather than straight-line propagation along the interface. The superior mechanical stability of FGFR at high loadings can be attributed to the coupled effects of interface and dispersion. First, the flat cross-section provides a specific surface area of 0.50 μm^−1^—about 1.6 times that of the circular fibers (0.31 μm^−1^), which enlarges the fiber–matrix contact area and improves the stress-transfer efficiency. Second, the flat geometry provides mechanical interlocking that suppresses interfacial debonding and fiber pull-out, as evidenced by the fracture morphologies in Figure 6. Third, the flat fibers maintain a preferential layered arrangement and a uniform dispersion in the PBT matrix even at 50 wt% (Figure 4d and Figure 5d), whereas the circular fibers entangle and agglomerate (Figure 6b); consistently, the geometry-specific agglomeration correction is much weaker for FGFR (*η*a = 0.750) than for CGFR (*η*a = 0.582) at the highest loading (Section 3.2).

The absence of significant flattening in the FGFR curve indicates that the reinforcement efficiency of the flat fibers has not yet saturated: the fiber–matrix interface remains below its stress-transfer limit, and the fiber volume fraction (0.340) is still well below the theoretical maximum packing fraction of the flat fibers (0.68, Equation (9)). The flat-fiber system has therefore not reached the agglomeration threshold at which strength deterioration begins for the circular-fiber system (*V_fmax_* = 0.526), suggesting that further property gains may still be achievable beyond 50 wt%, which will be explored in future work.

#### 3.1.3. Impact Toughness: Retained Energy Absorption Beyond 40 wt%

Figure 5b depicts the variation in notched impact strength of GF/PBT composites with glass fiber content (mean ± SD, *n* = 5; Table 3). For FGFR, the impact strength increases from 8.20 ± 0.88 kJ/m^2^ at 20 wt% to 12.33 ± 0.96 kJ/m^2^ at 30 wt%, reaches a maximum mean value of 15.74 ± 5.10 kJ/m^2^ at 40 wt%, and then decreases moderately to 13.07 ± 2.06 kJ/m^2^ at 50 wt%. For CGFR, the corresponding values are 6.04 ± 0.43, 9.52 ± 2.04, 14.08 ± 6.44, and 10.08 ± 0.75 kJ/m^2^, respectively. Pairwise Welch’s *t*-tests show that FGFR significantly outperforms CGFR at 20, 30, and 50 wt% (*p* = 0.003, 0.033, and 0.028, respectively), whereas the difference at 40 wt% is not significant (*p* = 0.66) owing to the large scatter shared by both systems at this loading. Within each system, one-way ANOVA followed by Tukey’s HSD test confirms a significant increase from 20 to 40 wt% (FGFR: *p* = 0.0033; CGFR: *p* = 0.0088), while the decrease from 40 to 50 wt% is not statistically significant for either system (*p* = 0.47 and 0.28, respectively); consistently, the 95% confidence intervals at 40 and 50 wt% overlap (Table 3). The scatter of both systems is largest at 40 wt%, identifying this region as a saturation–degradation transition zone in which fiber agglomeration begins to compete with reinforcement, rather than a sharply defined optimum [46]. Within this picture, fiber agglomeration progressively restricts the energy-absorption mechanisms (fiber pull-out, interfacial debonding, and matrix plastic deformation) beyond 40 wt%, and the decline from the maximum is milder for FGFR (−17.0%) than for CGFR (−28.4%).

To describe the impact energy absorption behavior of short-cut glass fiber-reinforced composites, this study adopts the fiber strain energy model [47]. According to this model, the impact energy absorption of composites consists primarily of two components: energy absorption due to matrix fracture and fiber-related energy absorption, which is expressed as [47]:

(11) $$\begin{array}{c} U_{c}=U_{x}V_{f}+(1-V_{f})U_{m} \end{array}$$

where *U_c_* represents the impact energy of the composite material, *U_x_* represents the fiber-related energy absorption term, *V_f_* represents the fiber volume fraction, and *U_m_* represents the matrix fracture energy. For short-fiber-reinforced systems, the fiber-related energy absorption term can be further expressed as:

(12) $$\begin{array}{c} U_{x}=U_{f}\left(\frac{L}{L+L_{c}}\right) \end{array}$$

In this equation, *L* is the average residual length of the glass fibers in the composite material, *L_c_* is the critical fiber length, and *U_f_* is the energy absorption associated with glass fiber fracture. By solving Equations (11) and (12) simultaneously, the theoretical expression for the impact energy of chopped glass fiber composites can be derived:

(13) $$\begin{array}{c} U_{c}=V_{f}U_{f}\left(\frac{L}{L+L_{c}}\right)+(1-V_{f})U_{m} \end{array}$$

The fracture energy of the PBT matrix was taken as 3.70 kJ/m^2^, and the energy absorption associated with glass fiber fracture is taken as 265 kJ/m^2^ [47]. These parameters are used together with the geometry-specific agglomeration factors in the corrected model (Equation (14)) below.

To account for the adverse effect of fiber–fiber interactions on the impact properties, the same agglomeration factor used in the tensile model (Equations (8) and (9), with the geometry-specific *V_fmax_*) is introduced into the fiber strain energy model for both systems and at all loadings, yielding the modified impact-energy model:

(14) $$U_{c}=\eta_{a}V_{f}U_{f}\frac{L}{L+L_{c}}+(1-V_{f})U_{m}$$

Substituting the above parameters into Equation (14) yields impact strengths of 9.21, 11.74, 13.41, and 13.24 kJ/m^2^ for the circular glass fiber/PBT composites and 13.09, 17.92, 22.21, and 25.01 kJ/m^2^ for the flat glass fiber/PBT composites at 20, 30, 40, and 50 wt%, respectively. The agglomeration factor is thus applied uniformly to both systems and at all loadings in both the tensile and the impact models, so that any residual mismatch between prediction and experiment can be attributed to physical mechanisms rather than to an asymmetric model treatment.

As shown in Figure 5b, the model reproduces the experimentally observed rise in the impact strength up to 40 wt% for both systems as well as the strength maximum of the CGFR system at 40 wt%. At 50 wt%, the model overpredicts both systems, and two points should be noted. First, the experimental decrease in FGFR between 40 and 50 wt% is itself not statistically significant (*p* = 0.33), i.e., the measured FGFR curve is essentially a plateau rather than a confirmed decline. Second, the continued increase predicted for FGFR arises because the mild geometry-specific correction (ηa = 0.750) is outweighed by the growing fiber term; the mean-field agglomeration factor, derived from a random-packing limit, cannot capture the sharp onset of local fiber clustering in the saturation–degradation transition zone identified above, and therefore overpredicts both systems at the highest loading. Additional deviations arise because the model idealizes fiber length, interfacial interactions, and fiber load-bearing efficiency, assuming that all glass fibers participate fully in stress transfer and impact-energy absorption; in actual composites, chopped glass fibers undergo further shear fracture during melt processing and injection molding and may exhibit non-uniform orientation, interfacial defects, and insufficient matrix coverage, which reduce the proportion of effective load-bearing fibers.

As shown in Figure 6e–h, flat glass fibers possess a larger specific surface area and more complex geometric features than conventional circular fibers. This increases the contact area between fibers and the PBT matrix and improves the interfacial adhesion strength by mechanical interlocking. The synergistic effect of several energy-dissipating mechanisms, including interfacial delamination, fiber pull-out, and matrix plastic deformation, effectively arrests crack initiation and propagation, thereby enhancing impact resistance. In circular fiber systems with high filler content, fiber agglomeration and incomplete matrix wetting are very likely because of random orientation and disordered distribution, leading to stress concentration and reduced toughening. In contrast, FGFR exhibits superior impact properties across all fiber contents, owing to the structural advantages of flat glass fibers and excellent interfacial bonding.

### 3.2. Suppressed Interfacial Polarization for Superior Dielectric Properties

**Figure 7:**
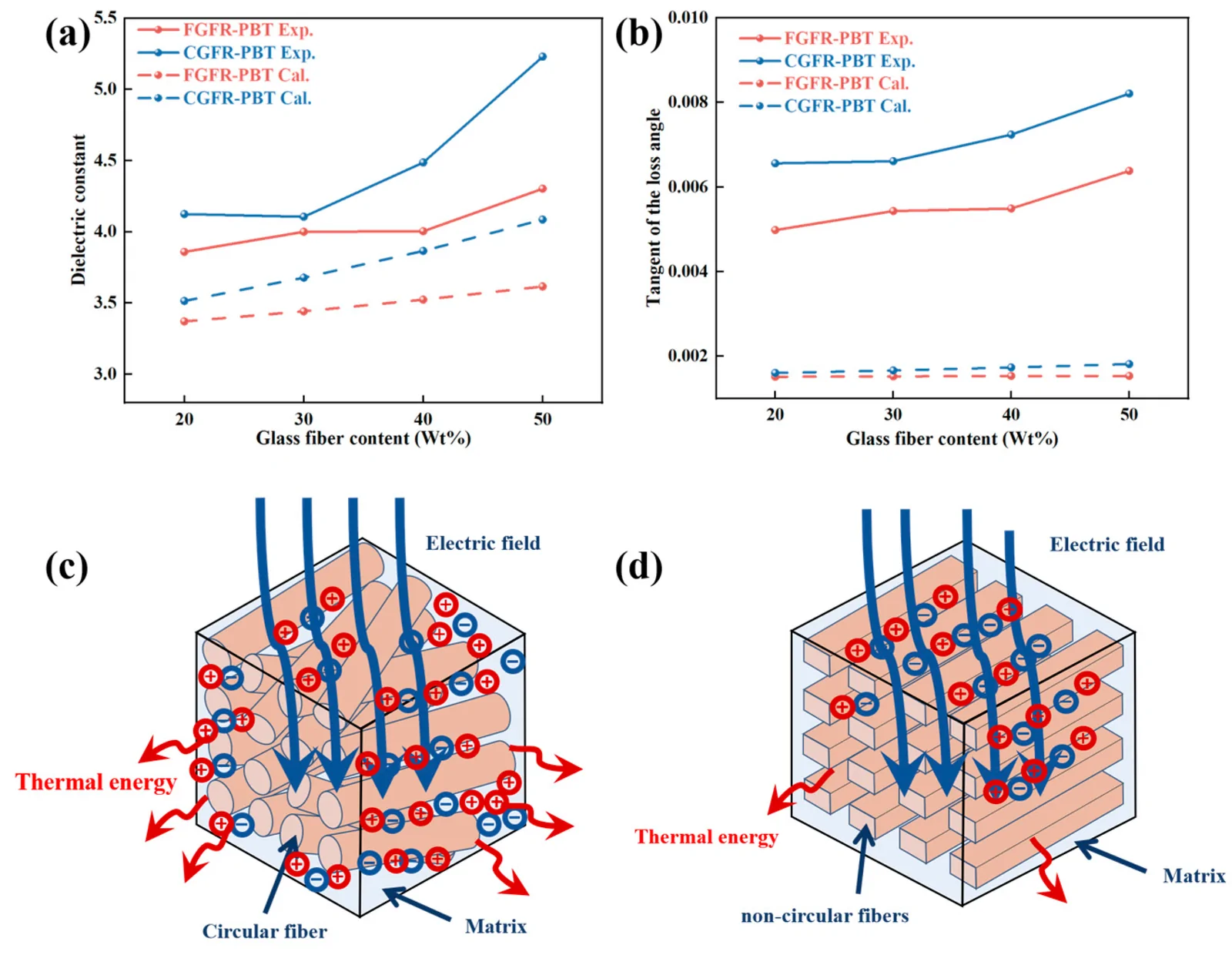
Dielectric properties and microstructural mechanisms of GF/PBT composites: (**a**) dielectric constant; (**b**) dielectric loss tangent; (**c**) schematic illustration of the dielectric loss mechanism in a system with circular glass fibers; (**d**) schematic illustration of the dielectric loss mechanism in a system with flat glass fibers.

The dielectric properties of glass-fiber-reinforced composites are primarily determined by matrix resin characteristics and glass fiber type [48]. As shown in Figure 2, the flat glass fibers themselves exhibit a lower dielectric constant (4.38 at 10 GHz) and loss tangent (0.0016) compared with typical E-glass fibers (εr ≈ 6.2, tanδ ≈ 0.0026). This intrinsic advantage, combined with the preferential layered arrangement of the flat fibers and their uniform interfacial distribution in the PBT matrix, contributes to the lower εr and tanδ of FGFR composites. Figure 7a,b show how the dielectric constant (εr) and dielectric loss tangent (tanδ) of glass fiber-reinforced PBT composites change with glass fiber content. As glass fiber content increases from 20 to 50 wt%, both εr and tanδ show an upward trend in FGFR and CGFR systems. In terms of εr, FGFR showed an 11.5% increase from 3.859 at 20 wt% to 4.303 at 50 wt%, while CGFR showed a 26.8% increase from 4.125 to 5.23. At comparable glass fiber content conditions, FGFR has a lower εr than CGFR. Both composites exhibited a gradual increase in tanδ with increasing glass fiber content, but FGFR maintained lower tanδ than CGFR across all fiber contents. At fiber contents of 20, 30, 40, and 50 wt%, the tanδ of FGFR was lower than that of CGFR by 24.1%, 17.8%, 24.1%, and 22.2%, respectively. Figure 7 shows the dependence of εr and tanδ in glass fiber on fiber content. In the frequency range of 0.6–10 GHz, εr exhibits a gradual decline from approximately 4.48 to 4.38, while tanδ increases from 0.011 to 0.016. The changes in the dielectric constant and the loss tangent are relatively slow over the whole range.

To further elucidate the influence of fiber shape on dielectric properties, this study introduces the Maxwell–Garnett (M–G) effective medium theory and combines it with shape correction factors to describe the localized polarization behavior of the reinforcing phase. This model accounts for the moderating effect of inclusion geometry on the depolarization field and is therefore more suitable for analyzing the relationship between fiber shape and dielectric properties in short-fiber-reinforced polymer composites [49]. According to the effective medium theory, the equivalent dielectric constant of a composite material can be expressed as:

(15) $$\varepsilon_{c}=\varepsilon_{m}\cdot \frac{\varepsilon_{f}+n\varepsilon_{m}+nV_{f}(\varepsilon_{f}-\varepsilon_{m})}{\varepsilon_{f}+n\varepsilon_{m}-V_{f}(\varepsilon_{f}-\varepsilon_{m})}$$

Here, *n* is the shape correction factor, used to describe differences in depolarization effects caused by the geometric morphology of the reinforcing phase. The shape factor n was set to 2.0 for circular fibers [50] and 3.5 for flat fibers based on their width-to-thickness ratio of 4:1 [51,52]. Regarding parameter selection, *ε_m_* is set to 3.25, *ε_f_* for circular glass fibers is 6.2, and the dielectric constant for flat glass fibers is taken from the 10 GHz test results in this study as 4.38. These parameters are substituted into the theoretical model for calculation, and the resulting theoretical values are compared and analyzed against the experimental results.

The dielectric loss tangent (*tanδ*) reflects the ratio of dielectric loss to energy storage capacity in a material under an alternating electric field; it essentially arises from the coupled effects of multiple mechanisms, including dipole orientation polarization, interfacial polarization, and conductive loss [53]. In fiber-reinforced polymer composites, *tanδ* is determined not only by the intrinsic losses of the matrix and reinforcing phases but is also significantly influenced by the structural state and carrier accumulation behavior in the interfacial region [54]. To further establish a quantitative relationship between the dielectric properties of glass-fiber-reinforced PBT composites and their compositional parameters, this study employs the logarithmic mixing rule to analyze the complex dielectric constant of the composite system. For a two-phase composite system, the complex dielectric constant can be expressed as:

(16) $$\varepsilon^{*}=\varepsilon \prime -j\varepsilon \prime \prime$$

Here, *ε*′ is the real part of the permittivity, which reflects a material’s ability to store electric field energy; *ε*″ is the dielectric loss factor (imaginary part), which characterizes a material’s ability to dissipate electromagnetic energy. Based on Lichtenecker’s logarithmic mixing model [55], the real and imaginary parts of the permittivity of a composite material satisfy the following volume fraction-weighted relationships, respectively:

(17) $${\ln\varepsilon }^{\prime \prime }=V_{f}\ln\varepsilon_{f}^{\prime \prime }+V_{m}\ln\varepsilon_{m}^{\prime \prime }$$

(18) $${\ln\varepsilon }^{\prime }=V_{f}\ln\varepsilon_{f}^{\prime }+V_{m}\ln\varepsilon_{m}^{\prime }$$

Here, *ε*′*_c_* and *ε*″*_c_* represent the real and imaginary parts of the dielectric constant of the composite material, respectively; *ε*′*_f_* and *ε*″*_f_* represent the real and imaginary parts of the dielectric constant of the glass fibers, respectively; *ε*′*_m_* and *ε*″*_m_* represent the real and imaginary parts of the dielectric constant of the PBT matrix, respectively. Applying an exponential transformation to Equations (17) and (18) yields the dielectric loss factor and the real part of the dielectric constant, respectively:

(19) $$\varepsilon^{\prime \prime }=\exp\left(V_{f}\ln\varepsilon_{f}^{\prime \prime }+V_{m}\ln\varepsilon_{m}^{\prime \prime }\right)$$

(20) $$\varepsilon^{\prime }=\exp\left(V_{f}\ln\varepsilon_{f}^{\prime }+V_{m}\ln\varepsilon_{m}^{\prime }\right)$$

According to the definition of the dielectric loss tangent:

(21) $$\tan\delta =\frac{\varepsilon^{\prime \prime }}{\varepsilon^{\prime }}$$

Then, the imaginary part of the dielectric loss can be expressed as:

(22) $$\varepsilon^{\prime \prime }=\varepsilon^{\prime }\tan\delta$$

Experimentally determined expression for the dielectric loss tangent:

(23) $$\tan\delta_{\exp}=\frac{\varepsilon_{c}^{\prime \prime }}{\varepsilon_{c}^{\prime }}$$

Substituting Equations (19) and (20) into Equation (22) yields a theoretical model for predicting the dielectric loss tangent of composite materials:

(24) $$\tan\delta_{\exp}=\frac{\exp\left(V_{f}\ln\varepsilon_{f}^{\prime \prime }+V_{m}\ln\varepsilon_{m}^{\prime \prime }\right)}{\exp\left(V_{f}\ln\varepsilon_{f}^{\prime }+V_{m}\ln\varepsilon_{m}^{\prime }\right)}$$

Using the properties of exponential functions, this can be further simplified to:

(25) $$\tan\delta_{\exp}=\exp\left[V_{f}\ln\left(\frac{\varepsilon_{f}^{\prime \prime }}{\varepsilon_{f}^{\prime }}\right)+V_{m}\ln\left(\frac{\varepsilon_{m}^{\prime \prime }}{\varepsilon_{m}^{\prime }}\right)\right]$$

The permittivity values ε_f_ of the circular and flat glass fibers are 6.13 and 4.38, respectively. The dielectric loss tangent is 0.0026 for the circular glass fibers and 0.0016 for the flat glass fibers, the latter measured at 10 GHz in this study. These parameters were substituted into the theoretical equations, and the calculated dielectric constant and loss tangent were compared with the experimental results in Figure 7a,b, respectively, to further analyze the influence of different fiber cross-sectional morphologies on the dielectric properties of the composite material.

The established model effectively captures the influence of varying glass fiber content on the dielectric properties of the composite material. Both the theoretical predictions and the experimental results show a gradual increase with rising fiber content, indicating that the model is reliable in describing these trends. For the dielectric constant, the theoretical model generally yields values lower than the experimental measurements. This is primarily because the model assumes ideal, uniform fiber dispersion and regular orientation, whereas actual composites exhibit non-uniform fiber orientation, interfacial polarization, localized agglomeration, and microscopic defects that increase energy dissipation—factors not fully accounted for in the simplified theoretical model. Increasing fiber content increases the number of interfaces and the polarization activity of fiber-reinforced polymer composites, leading to an increase in εr [8]. Interfacial polarization and dipole relaxation are accompanied by increased energy loss, leading to a rise in tanδ [56]. At the same fiber content, the dielectric properties of the composites are strongly affected by fiber morphology and interfacial structure. Compared with circular glass fibers, flat glass fibers tend to adopt a preferential layered arrangement, with their wide faces oriented parallel to the specimen surface, together with a more uniform interfacial distribution within the PBT matrix. This layered fiber arrangement and continuous interfacial structure alleviate local electric-field distortion, reduce disordered charge accumulation and abnormal charge aggregation at the fiber–matrix interface, and effectively suppress excessive interfacial polarization. As a result, the flat glass fiber-reinforced PBT composites exhibit a lower dielectric constant and dielectric loss. As shown in Figure 7c,d, the flat fibers form a more stable interface bond with the matrix, which suppresses delamination and defect formation. This limits repeated migration of interfacial charges and the polarization relaxation process. Therefore, FGFR has relatively small values of εr and tanδ, indicating more stable dielectric properties.

### 3.3. Enhanced Melt Flowability Under High Filler Loadings

**Figure 8:**
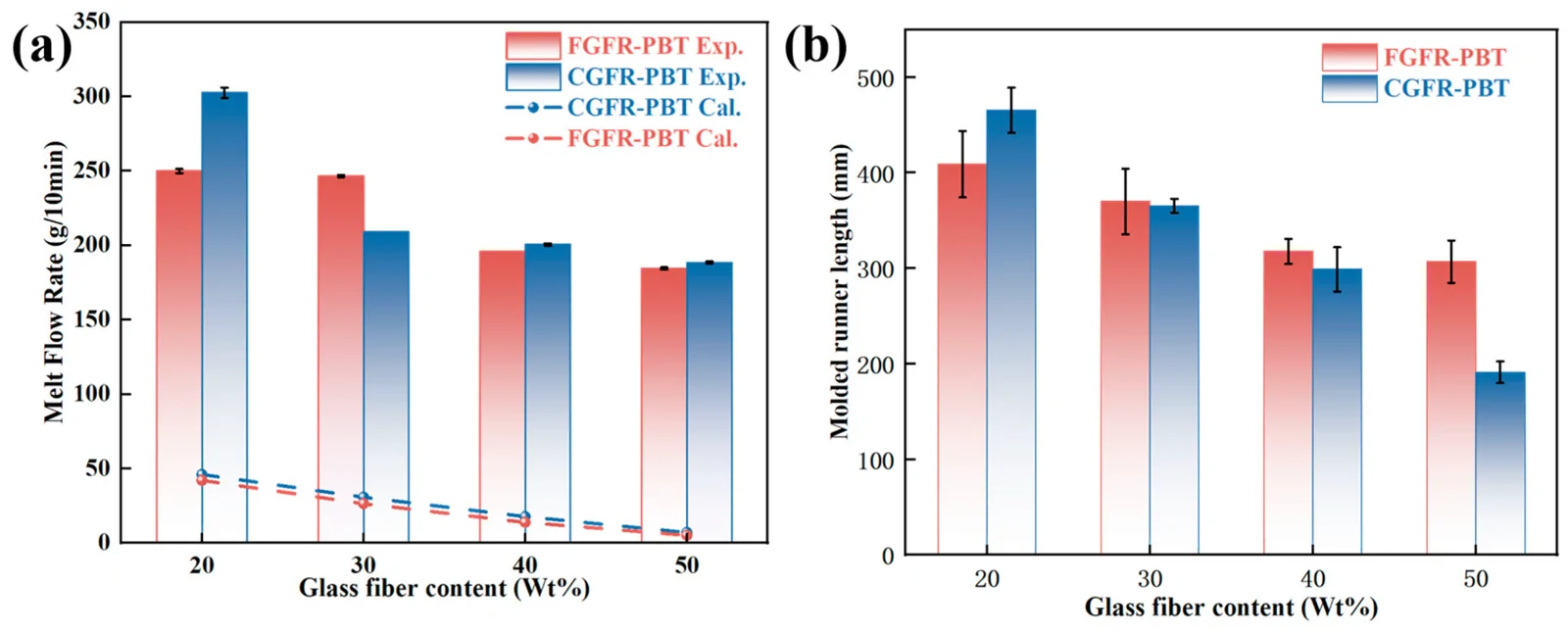
(**a**) Melt flow index of wave-transparent, flat glass fiber-reinforced PBT composites and reference samples. (**b**) Molding flow channel length of wave-transparent, flat glass fiber-reinforced PBT composites and reference samples.

The melt flow rate (MFR) is an important indicator of the processability of thermoplastic polymer composites; test results primarily reflect the material’s melt flow behavior at specific temperature and load conditions [57]. Figure 8a depicts the MFR value trends for the flat and circular glass fiber systems as a function of fiber content. The MFR of FGFR decreases with increasing fiber content. The MFR of FGFR reaches its peak at 20 wt% fiber content. The MFR decreases slightly as the glass fiber content approaches 30 wt%. As the glass fiber content increased from 20 to 50 wt%, the melt flow rate of the FGFR system decreased by about 35.5%, whereas that of CGFR decreased by about 60.5%. Figure 8b depicts the mold flow channel lengths for flat and circular systems with varying glass fiber contents. As the glass fiber content increased from 20 wt% to 50 wt%, both systems’ mold flow channel lengths decreased significantly. At a glass fiber content of 20 wt%, the flow channel length of CGFR was slightly longer than that of FGFR. However, when the glass fiber content increased to 40 wt% or higher, the flow channel length of FGFR was significantly longer than that of CGFR, with the most pronounced difference at 50 wt%.

To further elucidate the mechanisms underlying differences in the flow properties of irregular glass fiber-reinforced PBT composites and to establish a quantitative relationship between fiber cross-sectional geometry and melt processing behavior, this study analyzes the effects of fiber filling on melt rheological behavior. To further elucidate the mechanisms underlying differences in the flow properties of irregular glass fiber-reinforced PBT composites and to establish a quantitative relationship between fiber cross-sectional geometry and melt processing behavior, this study analyzes the effects of fiber filling on melt rheological behavior. Combining the melt flow characteristics of polymer composites with the Krieger-Dougherty (K-D) viscosity theory for suspension systems, this study established an MFR prediction model for glass fiber-reinforced PBT composites [58]. For particle-filled systems, the relative viscosity can be expressed as:

(26) $$\eta_{r}=\frac{\eta }{\eta_{0}}$$

Here, *η* is the apparent viscosity of the composite system, and *η*_0_ is the viscosity of the pure matrix resin. According to the Krieger–Dougherty model [59], the relative viscosity of the composite system is related to the filler volume fraction as follows:

(27) $$\eta_{r}={\left(1-\frac{\phi }{\phi_{m}}\right)}^{-[\eta ]\phi_{m}}$$

where *ϕ* represents the fiber volume fraction, and *ϕ_m_* represents the maximum packing volume fraction; *[η]* represents the intrinsic viscosity, which is used to characterize the influence of fiber geometric features on the rheological behavior of the composite system, and its value is closely related to the fiber aspect ratio. Based on calculations using the average length and diameter parameters of circular glass fibers, their intrinsic viscosity is approximately 4.3. For flat glass fibers, the intrinsic viscosity was estimated as approximately 5.0 using an equivalent aspect ratio correction [60].

According to the theory of aggregate rheology, under identical conditions of load, temperature, and capillary dimensions, the melt flow rate (MFR) is approximately inversely proportional to the melt viscosity:

(28) $$\mathrm{MFR}\propto \frac{1}{\eta }$$

Therefore, it can be written as:

(29) $$\frac{\mathrm{MFR}}{\mathrm{MF}R_{0}}=\frac{\eta_{0}}{\eta }$$

where *MFR*_0_ represents the melt flow rate of pure PBT resin, which is 80 g/10 min. By substituting the defined relative viscosity into the Krieger–Dougherty model and rearranging the terms, the following predictive model for the melt flow rate of glass-fiber-reinforced composites was obtained:

(30) $$\mathrm{MFR}=\mathrm{MF}R_{0}{\left(1-\frac{\phi }{\phi_{m}}\right)}^{\left[\eta \right]\phi_{m}}$$

By substituting the relevant model parameters into the above equations, the melt flow rates of the composites at different fiber volume fractions were calculated, and the corresponding prediction curves were plotted to analyze the effect of fiber content on melt flow behavior. The Krieger–Dougherty model captures the trend of decreasing MFR with increasing fiber content for both systems. However, the model substantially underestimates absolute MFR values because it assumes rigid, non-interacting particles and does not account for shear-thinning, wall slip, or lubrication effects during melt flow. Despite this quantitative limitation, the model correctly predicts the relative difference between the two systems—namely, the smaller MFR decline for FGFR at high loadings. The rheological properties of chopped glass fiber-reinforced thermoplastics depend not only on filler content but also on fiber cross-sectional shape, specific surface area, and orientation evolution within the flow field [61]. The flat fiber systems showed more stable rheological behavior in both MFR and molding flow length tests. Compared with the circular glass fiber system, the flat system showed a longer molding flow path under high fiber loading, indicating that FGFR can effectively suppress premature cooling of the melt front during injection molding.

## 4. Conclusions

This work systematically investigated the mechanical, dielectric, and rheological properties of PBT composites reinforced with flat glass fibers (FGFR) and revealed their significant advantages over conventional circular glass fibers (CGFR), especially at high loadings. The main conclusions are as follows: (1) Flat glass fibers impart superior mechanical performance. At 50 wt% loading, FGFR achieves a tensile strength of 116 MPa (11.4% higher than CGFR) and a notched impact strength of 13 kJ/m^2^ (~30% higher). The enhanced reinforcement stems from the larger specific surface area (1.61 times that of circular fibers), which increases fiber–matrix interfacial contact. Microstructural analysis and the modified Kelly–Tyson and Halpin–Tsai models with a unified, geometry-specific agglomeration correction semi-quantitatively capture this behavior, confirming that flat fibers maintain good dispersion at high loadings and effectively avoid the performance cliff caused by fiber agglomeration in CGFR. (2) Flat glass fibers reduce the dielectric constant and dielectric loss. At 10 GHz and 50 wt% fiber content, FGFR exhibits a dielectric constant of 4.30 and a loss tangent of 0.014, which are 17.8% and 22.2% lower, respectively, than those of CGFR. The preferential layered arrangement and uniform interfacial distribution of flat fibers suppress interfacial polarization, as supported by the Maxwell–Garnett effective medium model. (3) Flat glass fibers provide better melt processability under high filler conditions. When the fiber content increases from 20 to 50 wt%, the melt flow rate of FGFR drops by only 35%, far less than the 60% drop for CGFR, and its mold flow channel length remains significantly longer at high loadings. The Krieger–Dougherty model with geometry-dependent intrinsic viscosity successfully describes this behavior, confirming that the flat cross-section reduces flow resistance in highly filled melts.

These findings establish a clear structure-property relationship for the current flat fiber/PBT system, paving the way for future exploration of this geometry-dependent strategy across a broader range of fiber aspect ratios and thermoplastic matrices.

## Figures and Tables

**Table 1:** Formulations for glass-fiber-reinforced PBT composites.

Group	Sample Name	Component Weight (wt%)		
		PBT	HL303N-M4	ECT303K
Experimental Sample	FGFR-G20	80	20	-
	FGFR-G30	70	30	-
	FGFR-G40	60	40	-
	FGFR-G50	50	50	-
Comparison Sample	CGFR-G20	80	-	20
	CGFR-G30	70	-	30
	CGFR-G40	60	-	40
	CGFR-G50	50	-	50

Note: In addition to the components listed, every formulation contains the same processing-aid package of the commercial pellets, comprising an epoxy-type chain extender (typically 0.3–1.0 wt%), hindered-phenolic and phosphite antioxidants (typically 0.1–0.5 wt% in total), and an ester-based lubricant (typically 0.1–0.5 wt%); the exact additive recipes are proprietary to the pellet supplier (CPIC). Because this package is identical for all eight groups, the only experimental variables are the fiber type and content.

**Table 2:** Performance test specifications for glass-fiber-reinforced PBT composite materials.

Test Items	Test Sample	Reference Standard	Testing Equipment
Tensile Properties Test	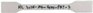	ASTM D638	Universal Testing Machine
Notch Impact Test	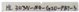	ASTM D256	Cantilever Beam Impact Testing Machine
Fiber Length Testing	-	GB/T 42923-2023	-
Dielectric Properties Testing	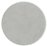	IEC 62562:2010	Vector Network Analyzer
Melt Index Test	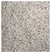	GB/T 3682.1-2018	Melt Index Tester
Flow Channel Length Test	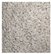	-	Injection molding machine
Cross-Section Morphology Characterization	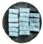	-	Field Emission Scanning Electron Microscope

## Data Availability

The original contributions presented in this study are included in the article. Further inquiries can be directed to the corresponding author.
